# Supplementary material for: Molecular Characterization and Pathogenesis of H6N6 Low Pathogenic Avian Influenza Viruses Isolated from Mallard Ducks (Anas platyrhynchos) in South Korea
Source: Viruses. 2022 May 8;14(5):1001. doi: 10.3390/v14051001 (PMC9143286; doi:10.3390/v14051001)
Supplement: Supplementary file 1 [file viruses-14-01001-s001.zip › viruses-1678586-supplementary.pdf]

viruses

MDPI

Supplementary material

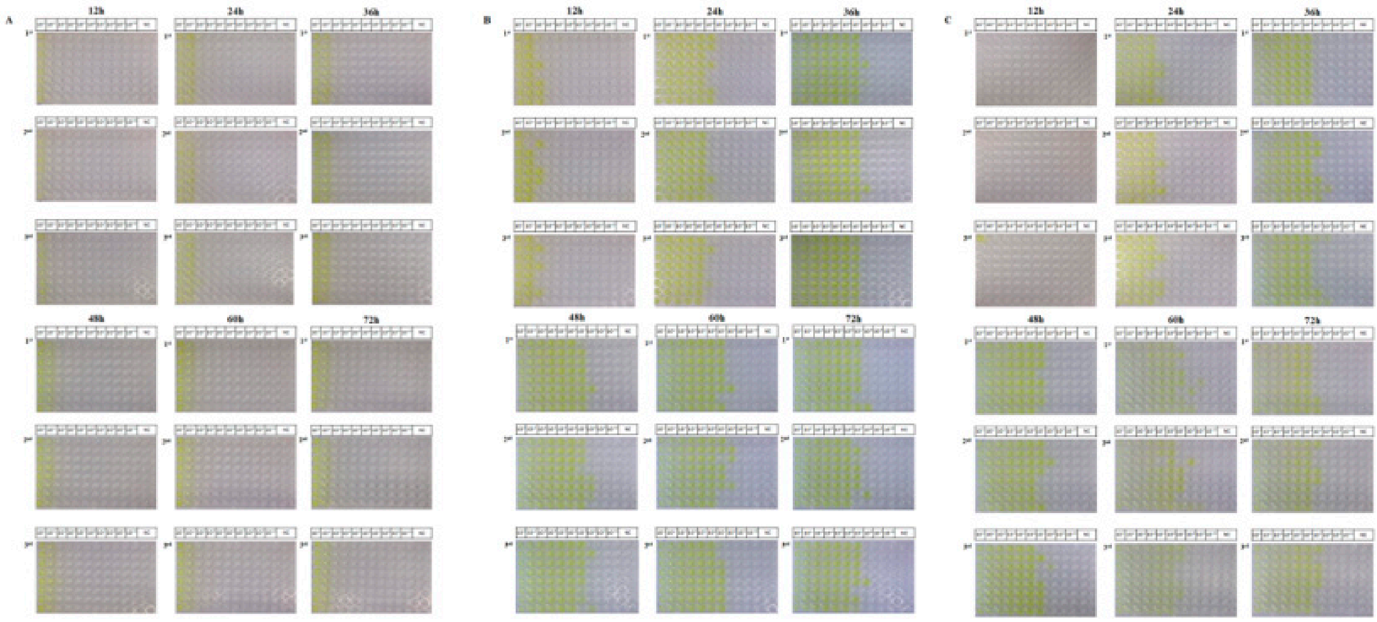

Figure S1. Raw ELISA data of the TCID50 assay for the detection of **(A)** KNU2019-48 (H6N6), **(B)** H1N1 (CA/04/09), and **(C)** H7N7 growth kinetics in MDCK cells.

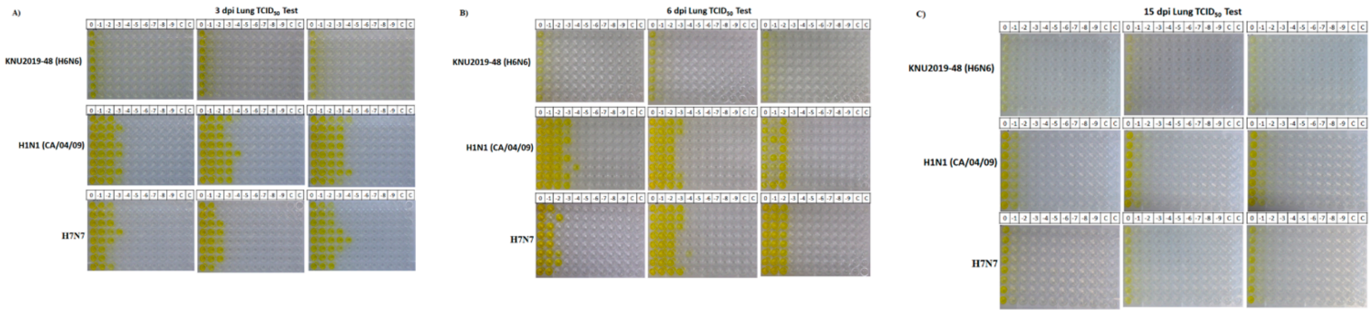

Figure S2. Raw ELISA data of the TCID50 assay for viral load shedding in lungs after **(A)** 3, **(B)** 6, and **(C)** 15 days post-infection.

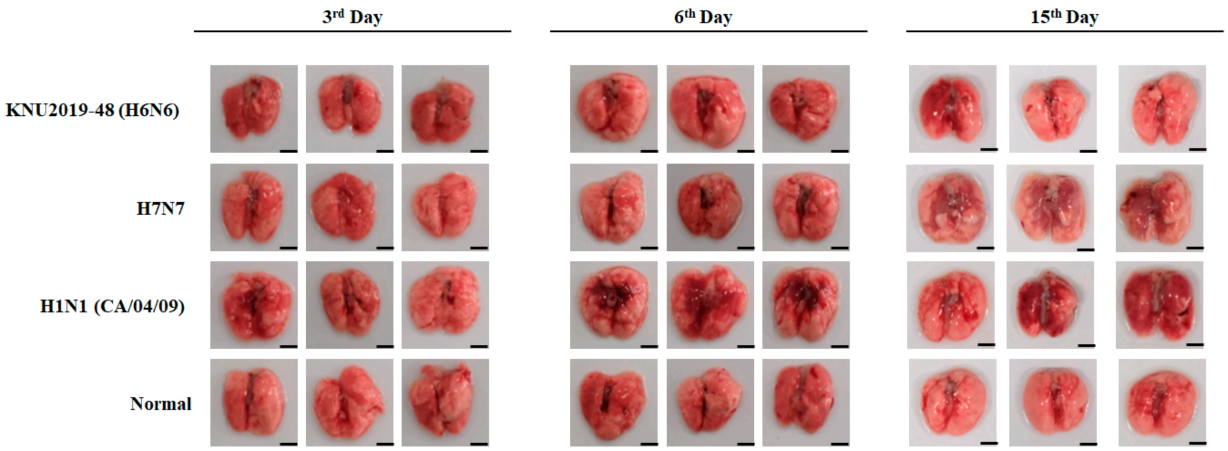

Figure S3. Lungs from normal and infected mice at 3, 6, and 15 days post-infection. Scale bar: 0.5 cm.

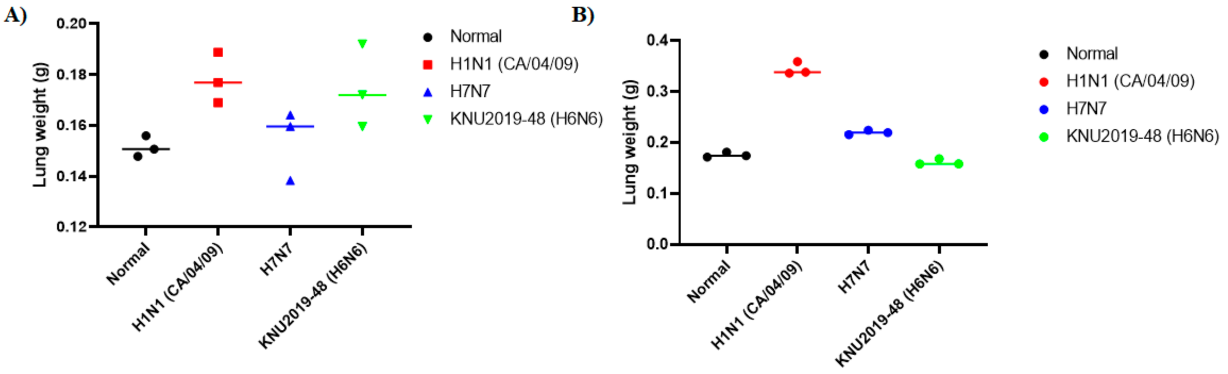

Figure S4. Lung weight at (**A**) 3 and (**B**) 15 days post-infection.
